# *Withania somnifera* as a potential candidate to ameliorate high fat diet-induced anxiety and neuroinflammation

**DOI:** 10.1186/s12974-017-0975-6

**Published:** 2017-10-12

**Authors:** Taranjeet Kaur, Gurcharan Kaur

**Affiliations:** Department of Biotechnology, Guru Nanak Dev University, Amritsar, Punjab 143005 India

**Keywords:** *Withania somnifera*, Neuroinflammation, Anxiety, Obesity, High fat diet, Inflammatory cytokines, Leptin, Insulin, Apoptosis

## Abstract

**Background:**

The epidemic of obesity has reached alarming levels in both developing and developed nations. Excessive calorie intake and sedentary lifestyle due to technological advancements are the main causal factors for overweight and obesity among the human population. Obesity has been associated with a number of co-morbidities such as hypertension, type 2 diabetes mellitus, cardiovascular diseases, and neurodegeneration and dementia. The progression of neurological disorders in obese subjects has been mainly attributed to neuroinflammation. *Withania somnifera* has been used in numerous Ayurvedic formulations owing to its wide array of health-promoting properties. The current study was designed to test the hypothesis whether dry leaf powder of *W*. *somnifera* has anxiolytic and anti-neuroinflammatory potential in diet-induced obesity.

**Methods:**

Young adult female rats were divided into four groups: low fat diet group (LFD) fed with regular chow feed, high fat diet group (HFD) fed with diet containing 30% fat by weight, low fat diet plus extract group (LFDE) fed with regular chow feed supplemented with dry leaf powder of *W*. *somnifera* 1 mg/g of body weight (ASH), and high fat diet plus extract group (HFDE) fed with diet containing 30% fat by weight and supplemented with ASH. All the animals were kept on respective feeding regimen for 12 weeks; following which, the animals were tested for their anxiety-like behavior using elevated plus maze test. The animals were sacrificed and used to study various inflammatory markers such as GFAP, Iba1, PPARγ, iNOS, MCP-1, TNFα, IL-1β, IL-6, and various markers of NF-κB pathway by Western blotting and quantitative real-time PCR. Serum levels of leptin, insulin and pro-inflammatory cytokines were also assayed.

**Results:**

ASH treated rats showed less anxiety levels as compared to HFD animals. At molecular level, ASH ameliorated the HFD-induced reactive gliosis and microgliosis and suppressed the expression of inflammatory markers such as PPARγ, iNOS, MCP-1, TNFα, IL-1β, and IL-6. Further, ASH ameliorated leptin and insulin resistance and prevented HFD-induced apoptosis.

**Conclusions:**

Dry leaf powder of *W*. *somnifera* may prove to be a potential therapeutic agent to attenuate neuroinflammation associated with obesity and may prevent its co-morbidities.

## Background

Overconsumption of palatable and high-calorie diet is responsible for chronic positive energy balance where the intake of energy exceeds energy expenditure, leading to extra body fat, weight gain, and obesity [1]. Obesity is now regarded as an inflammatory condition [2], which is characterized by increased production and secretion of pro-inflammatory cytokines such as TNFα and IL-6 [3]. Low-grade inflammation associated with obesity affects both peripheral organs and CNS [4] and has also been implicated in the pathology of various metabolic disorders such as cardiovascular diseases [5], type 2 diabetes mellitus [6], asthma [7], cancer [8], etc. The consumption of high-calorie diet has also been linked to various neuropsychiatric comorbidities, which include anxiety, mood disorders, binge eating, and cognitive impairments [9]. The pathology of these psychiatric conditions is intrigued by inflammatory processes originating from adipose tissue or gut microbiota [9].

The inflammatory signals emanating from periphery cross the blood–brain barrier and enter the CNS, where they cause neuroinflammation and also affect the neuroendocrine and neurotransmitter activity. The obese subjects have shown up to 30% more prevalence of depressive symptoms than the general age-matched population [10]. The depressive symptoms in rodent model of obesity have been attributed to the increases in BDNF and phospho-CREB [11]. Together, these changes have been shown to cause negative emotional states and depression-like symptoms in mice [11]. Several chemically synthesized anti-obesity medications such as amphetamines, rimonabant, and sibutramine have been used in clinical practice, which now have been withdrawn owing to their adverse side effects [12] during long-term therapy. The notable side effects of these drugs include adverse cardiovascular events, long-term dependency on these drugs, increased risk of anxiety, depression, and suicidal ideation [13]. The plant-based traditional medicinal systems continue to play an indispensable role in healthcare, particularly in the Indian subcontinent.

*Withania somnifera*, commonly known as Ashwagandha, is one of the most prominent medicinal plants mentioned in Ayurvedic scriptures. It has been classified as a “rasayana” (tonic) herb for its rejuvenating potential [14]. A commercially available root extract of *W*. *somnifera* has been shown to improve cardiorespiratory endurance in the healthy athletes after 8 and 12 weeks of consumption [15]. High concentration, full-spectrum root extract of *W*. *somnifera* has also been shown to improve resistance to stress in human subjects [16]. Root powder of the plant has been shown to reduce cholesterol, triglycerides, LDL, and VLDL in mild non-insulin-dependent diabetes mellitus and mild hypercholesterolemic human subjects [17] and to control hypertension [18]. A commercially available root extract of *W*. *somnifera* has been recently reported to be instrumental in management of body weight in obese adults under chronic stress by the reduction in serum levels of cortisol and physiological and psychological markers of stress thereby regulating the eating behavior [19]. In rodent model of type 2 diabetes mellitus, aqueous root extract of *W*. *somnifera* has been shown to prevent pancreatic β-cell damage and oxidative damage [20].

Our lab has recently shown the anti-neuroinflammatory potential of water extract from the leaves of *W*. *somnifera* in both in vitro [21] and in vivo [22] model systems. In continuation of earlier reports, the current study was designed to investigate the potential beneficial effects of dry leaf powder of *W*. *somnifera* on neuroinflammation and anxiety resulting from diet-induced obesity (DIO). In comparison to root powder, use of leaf powder is eco-friendly as there is no need to sacrifice the plant as well as it is easy and convenient to prepare. Obesity was induced by feeding the rats with 30% fat diet for 12 weeks, and in one group of animals, high fat diet was supplemented with the dry leaf powder of *W*. *somnifera*. Post regimen, the effect of co-supplementation of *W*. *somnifera* was studied on anxiety-like behavior induced by DIO and the animals were sacrificed to study the expression of marker proteins associated with reactive gliosis (Glial Fibrillary Acidic Protein (GFAP)) and inflammation (Iba1, PPARγ, TNFα, IL-1β, IL-6) in the hippocampus, piriform cortex (PC), and hypothalamus regions of the brain. The levels of pro-inflammatory cytokines, leptin, and insulin were assayed in the sera collected using ELISA-based assay. The expression of leptin receptor OB-Rb was also studied by Western blotting and quantitative real-time PCR. Further, the NF-κB pathway of inflammation was investigated to understand underlying molecular mechanisms of the effect of dry leaf powder of *W*. *somnifera* in ameliorating the impairments caused due to DIO.

## Methods

### Preparation of dry leaf powder of *W*. *somnifera*

The leaves were collected from the seed-raised *W*. *somnifera* plant growing at the herbarium of the Department of Botanical and Environmental Sciences, Guru Nanak Dev University, Amritsar. The plant was identified and authenticated by the plant taxonomist and was deposited for reference purpose with voucher specimen number 401-24/07/1982. The previous studies from our lab have used water extract from the leaves of *W*. *somnifera* plant (ASH-WEX) [21–23], but in the current study, we have used dry leaf powder of *W*. *somnifera* (ASH). The dose of ASH powder has been calculated based on previous in vivo reports from our lab [22, 24] using ASH-WEX (dry weight) at a dose of 140 mg/kg body weight of the animal. One milligram dry weight of ASH-WEX is equivalent to 6.80 mg of dry leaf powder, so the dose corresponds to 952 mg/kg body weight or ~ 1 g/kg body weight or 1 mg/g body weight. ASH-WEX has been standardized and characterized in the previous studies from our lab [24, 25].

### Experimental animals and administration of the extract

Wistar albino young female rats in the age group of 3–4 months and weighing 130–150 g were used for all the experiments. The animals were housed in the group of three animals per cage under controlled environmental conditions (temperature 25 ± 2 °C and light/dark cycle of 12:12 h) with *ad libitum* supply of food and water. Animal care and procedures were followed in accordance with the guidelines of Institutional Animal Ethical Committee. The animals were categorized randomly into four groups: low fat diet group (LFD) fed with regular chow feed (2.5–5% fat, 75–77.5% carbohydrates, 20% proteins as macronutrients), high fat diet group (HFD) fed with diet containing 30% fat by weight (30% fat, 50% carbohydrates, 20% proteins as macronutrients), low fat diet plus extract group (LFDE) fed with regular chow feed supplemented with ASH, and high fat diet plus extract group (HFDE) fed with diet containing 30% fat by weight and supplemented with ASH (*n* = 10 ± 1 animals per group). Regular chow feed and high fat diet were mixed with ASH at a concentration of 1 mg/g body weight of animal. All animals were kept on the respective dietary regimen for a period of 12 weeks. The body weight of animals was monitored every week. Daily food intake for animals of all groups was also recorded. LFDE group was included in our study to elucidate any additional benefits of ASH in rats kept on normal diet. Since no significant changes were observed in the LFDE group, only preliminary data has been presented for this group.

### Elevated plus maze test

The elevated plus maze (EPM) is a plus-shaped apparatus consisting of two opposing closed arms (50 cm × 10 cm × 40 cm), two opposing open arms (50 cm × 10 cm), and a central open area, elevated 50 cm above the ground. Individual rat from each group was placed in the central open area, facing one of the open arms at the beginning of the test. It was allowed to freely explore the maze for the duration of 5 min. The whole experiment was set up in dim light conditions and without any human disturbance. The behavior of each rat was recorded in a video camera. The whole apparatus was wiped with 70% ethanol before placing the next rat in the maze. Video recording of each rat was later analyzed for the number of crossings, number of entries, total time spent in each of the arms, and total number of head dips. An entry into an arm was counted only when all the four limbs of the rat were in that arm. A crossing was defined as the entry of a rat from an arm directly to its opposing arm. Also, total time spent in open and closed arms was recorded. A head dip was defined as looking down from the edge of an open arm or from central open area.

After subjecting the animals to EPM test, animals were sacrificed and used for immunohistochemical staining (*n* = 3–4 each group), Western blot analysis (*n* = 3–4 each group), and quantitative real-time PCR analysis (*n* = 3–4 each group).

### Expression study of GFAP by immunohistochemical staining

For the expression study of GFAP, the animals (*n* = 3 each group) were perfused transcardially with 4% paraformaldehyde (PFA) in phosphate-buffered saline (PBS, 0.1 M). Brains were dissected out and stored in fixative (4% PFA) overnight at 4 °C. Subsequently, the brains were cryopreserved in grades of sucrose from 10–30% for 24 h each at 4 °C. Coronal sections of 35 μm thickness were cut using a cryostat microtome (Thermo Scientific Shandon, Waltham, MA, USA). The sections were collected in a petri dish and were given three washes with 0.1 M PBS pH 7.4, 15 min each, followed by incubation with 2% H_2_O_2_ for 15 min. The sections were then incubated with 0.3% Triton X-100 in 0.1 M PBS for 30 min for permeabilization followed by washing with phosphate-buffered saline with 0.1% Triton X-100 (0.1% PBST) thrice for 5 min each and incubation in 5% normal goat serum (NGS) in 0.1 M PBS for 30 min. The sections were then incubated with an antibody, i.e., rabbit IgG anti-GFAP (1:500) (Sigma Aldrich, St. Louis, MO, USA) in 5% NGS for 48 h at 4 °C. Sections were washed with 0.1% PBST thrice for 5 min each and incubated with a secondary antibody, i.e., goat anti-rabbit IgG-biotin conjugate (1:400) (Sigma Aldrich, St. Louis, MO, USA) in 0.1% PBST for 2 h at room temperature. Sections were washed with 0.1% PBST thrice for 5 min each. Further, the sections were incubated with ExtrAvidin-Peroxidase (1:400) (Sigma Aldrich, St. Louis, MO, USA) in 0.1% PBST for 2 h at room temperature, followed by diaminobenzidine (DAB) (Sigma Aldrich, St. Louis, MO, USA) for 15–20 min. Sections were mounted on the glass slide and air-dried overnight followed by dehydration through grades (50, 70, 90, and 100%) of ethanol. Tissue sections were then coverslipped using DPX permanently. Images of the sections were captured using Life Technologies EVOS FL microscope. To ensure the specificity of GFAP immunostaining, negative control was used in which the incubation with primary antibody (rabbit IgG anti-GFAP) was omitted.

### Expression study of proteins by western blotting

Animals (*n* = 3–4 each group) were anaesthetized with sodium thiopentone injection and sacrificed by decapitation. Brains were dissected to isolate PC, hippocampus, and hypothalamus regions, which were homogenized in a buffer (1X Tris buffered saline, dithiothreitol (DTT), Na_3_VO_4_, protease inhibitor) using a tissue homogenizer at 4 °C. The homogenate was centrifuged at 10,000*g* for 10 min at 4 °C. The supernatant was separated and used for protein estimation by Bradford method. Equal quantity of protein from each sample was mixed with 6X sample buffer (0.25 M Tris-HCl (pH 6.8), 20% glycerol, 4% sodium dodecyl sulfate (SDS), 10% β-mercaptoethanol, and 1 mg bromophenol blue) and boiled for 2–3 min. 30-50 μg of protein sample was loaded in each well for electrophoretic separation by SDS-PAGE in 10–15% gels, followed by transfer onto a PVDF membrane (Hybond-P, GE Healthcare, USA) using the semidry TE70 PWR system (Amersham Biosciences, USA). Subsequently, the membrane was blocked (5% skimmed milk in 0.1% TBS-Tween 20) and immediately incubated with respective primary antibody (mouse anti-GFAP (1:2000); mouse anti-TNFα (1:1000); mouse anti-IL-1β (1:1000); mouse anti-IL-6 (1:1000); mouse anti-Bcl-xL (1:2000); rabbit anti-AP-1 (1:2000) (Sigma Aldrich, St. Louis, MO, USA); mouse anti-Iba1 (1:1000) (EMD Millipore, CA, USA); rabbit anti-OB-Rb (1:1000); mouse anti-PPARγ (1:1000) (Santa Cruz Biotechnology, Dallas, TX, USA); rabbit anti-phospho IKKα/β (1:1500); mouse anti-IKKα (1:1500); mouse anti-IKβα (1:1500); rabbit anti-NF-κB (1:1500) (NF-κB Pathway Sampler Kit, Cell Signaling Technology, MA, USA)) at 4 °C overnight, followed by three washings with 0.1% TBST and incubation with respective secondary antibody (goat anti-mouse for GFAP, TNFα, IL-1β, IL-6, Bcl-xL, Iba1, PPARγ, IKKα, and IKβα and goat anti-rabbit for AP-1, OB-Rb, phospho IKKα/β, and NF-κB) conjugated with horse radish peroxidase (HRP) (1:5000) (Merck Millipore, USA) for 2 h at room temperature. The membranes were also probed with anti-α-tubulin antibody (1:5000) (Sigma Aldrich, St. Louis, MO, USA) as an internal control for protein loading. Immunoreactive bands on membrane were visualized by ECL Prime Western Blotting Detection Reagent (GE Healthcare, Little Chalfont, UK) according to the manufacturer’s instructions using Image Quant LAS 4000 system (GE Healthcare, Little Chalfont, UK). Intensity analysis was done using AlphaEase FC software (Alpha Innotech, CA, USA). Change in expression of proteins of interest was taken as the average of integrated density values (IDV) obtained from at least three independent experiments.

### mRNA expression analysis by quantitative real-time PCR

Total RNA was extracted from the tissues using TRI reagent (Sigma Aldrich, St. Louis, MO, USA) according to the manufacturer’s instructions. For cDNA synthesis, 5 μg of RNA, 1 μL of 250 ng random hexamers (Life Technologies, Carlsbad, CA, USA), 1 μL of 10 mM dNTP mix (Sigma Aldrich, St. Louis, MO, USA), and nuclease-free sterile water up to 12 μL were added. The mixture was heated to 65 °C for 5 min and quick-chilled on ice. 4 µL of 5X first-strand buffer, 2 μL of 0.1 M DTT, and 1 μL of recombinant ribonuclease inhibitor (40 units/μL) (Life Technologies, Carlsbad, CA, USA) were added and incubated at 37 °C for 2 min. Then, 1 μL (200 units) of M-MLV reverse transcriptase (Life Technologies, Carlsbad, CA, USA) was added for 20 μL reaction volume. The mixture was kept at 25 °C for 10 min and at 37 °C for 50 min followed by inactivation of reaction by heating at 70 °C for 15 min in a thermal cycler. 50 µg of cDNA was then used to amplify gene of interest using Step One Plus Real Time PCR system (Applied Biosystems, Foster City, CA, USA). Each 5 μL reaction mixture comprised of 2.5 μL of 2X Power-SYBR Green Master Mix (Applied Biosystems, Foster City, CA, USA), 1 μL of 20X pre-designed Primer mix (Integrated DNA Technologies, Coralville, IA, USA), 1 μL of water, and 0.5 μL (50 μg) cDNA. GAPDH was used as an endogenous control for each gene of interest. The relative gene expression of each gene was calculated by “Livak method” [26] and represented as ${2}^{-\Delta \Delta {\mathrm{C}}_{\mathrm{T}}}$.

### Estimation of levels of pro-inflammatory cytokines, leptin, and insulin in serum by ELISA

The levels of pro-inflammatory cytokines TNFα, IL-1β, and IL-6 along with leptin and insulin were assayed using sandwich ELISA kits. The kits for inflammatory cytokines were procured from Sigma Aldrich (St. Louis, MO, USA) and for leptin and insulin from EMD Millipore Corporation (USA). The estimations were done based on colorimetric detection according to manufacturer’s protocol. The concentrations of TNFα, IL-1β, IL-6, leptin, and insulin in each sample were calculated using kit provided standards and Sigma Plot software.

### Statistical analysis

Values are expressed as mean ± SEM from at least three independent experiments. The Sigma Stat for Windows (version 3.5) was used to analyze the results by one-way ANOVA (Holm-Sidak post hoc method), in order to determine the significance of the means. Two-way ANOVA was performed on EPM test data to determine the level of significance for parameters within the group and between the groups. Values with *p* value ≤ 0.05 were considered as statistically significant.

## Results

### ASH was instrumental in management of body weight

**Fig. 1:**
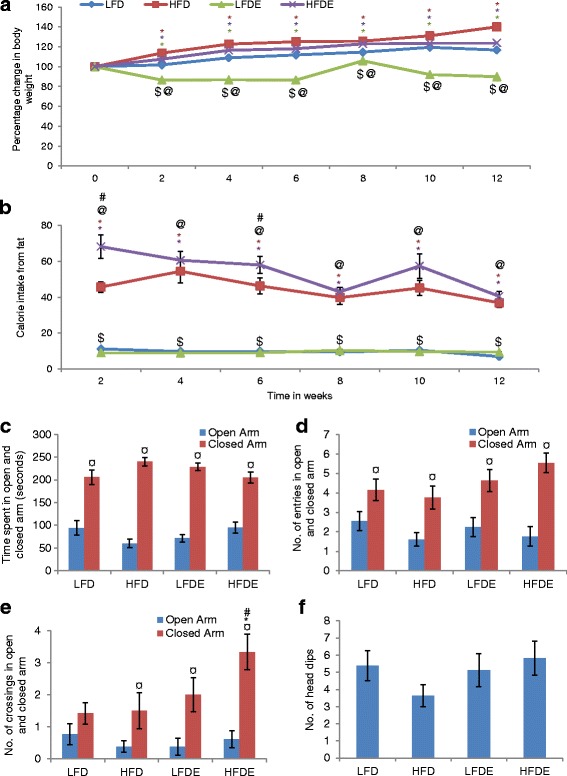
ASH supplementation maintained body weight and suppressed anxiety-like behavior in HFD-induced obese rats. **a** Line graph representing the percentage change in body weight among different groups of animals over the period of 12 weeks (*n* = 10 ± 1 each group). **b** Histogram represents average time spent by the animals in open and closed arms of elevated plus maze. Among the four groups, HFD animals spent maximum time in the closed arm. The exploratory activity of animals of each group is shown by the number of entries (**c**) and number of crossings (**d**) in open and closed arms. HFDE animals showed more exploratory activity than the other groups. **e** Histogram represents the number of head dips by the animals of each group. Values are expressed as mean ± SEM. * *p* ≤ 0.05 LFD versus HFD, LFDE, and HFDE rats; ^#^ *p* ≤ 0.05 HFD versus HFDE rats; ^$^ *p* ≤ 0.05 HFD versus LFDE rats; ^@^ *p* ≤ 0.05 LFDE versus HFDE rats; ^¤^ statistically significant difference within group, Holm–Sidak method after one-way ANOVA in **a**, **b** and **f** and two-way ANOVA in **c**, **d** and **e**

A gradual increase in the body weight was observed in the rats of LFD, HFD, and HFDE group over the period of 12 weeks. On the completion of respective regimen for 12 weeks, LFD and HFD rats weighed 16.7% and 40.1%, respectively, more than their initial weight (Fig. 1a). HFDE rats also weighed 23.9% more than their initial weight, but on the contrary, LFDE rats showed 10.2% reduction of body weight after 12 weeks of feeding ASH-supplemented normal chow diet. Further, the amount of calories from fat was also calculated for each week. Highest calorie intake was observed in the HFDE group over the period of 12 weeks (Fig. 1b), though it was reduced by 40.63% at the end of the 12th week as compared to initial intake. The HFD group also showed gradual reduction in calorie intake over the 12-week period. However, LFD and LFDE groups showed consistent calorie intake from fat during the respective regimens of 12 weeks.

### ASH suppressed anxiety-like behavior in diet-induced obese rats

Prior to the EPM test, the animals were not given any training. Among the four groups, HFD rats spent minimum time in the open arm and maximum time in the closed arm as compared to LFD, LFDE, or HFDE rats (Fig. 1c). The HFDE group showed EPM profile comparable to LFD group. Further, HFD animals also showed less number of entries in both open and closed arms as compared to LFD animals (Fig. 1d). The number of crossings in the open arm was also reduced in HFD animals as compared to LFD animals (Fig. 1e). This behavior was reversed in HFDE animals, which showed highest number of entries and significantly high number of crossings (*p* ≤ 0.05) into the closed arm. Further, the number of head dips, which is an indicator of anxiety in rodents, was also reduced in HFD animals as compared to LFD animals, while HFDE animals showed head dips comparable to LFD animals (Fig. 1f). Overall, the behavior of LFDE animals was comparable to LFD animals.

### ASH suppressed reactive gliosis and modulated inflammatory response

**Fig. 2:**
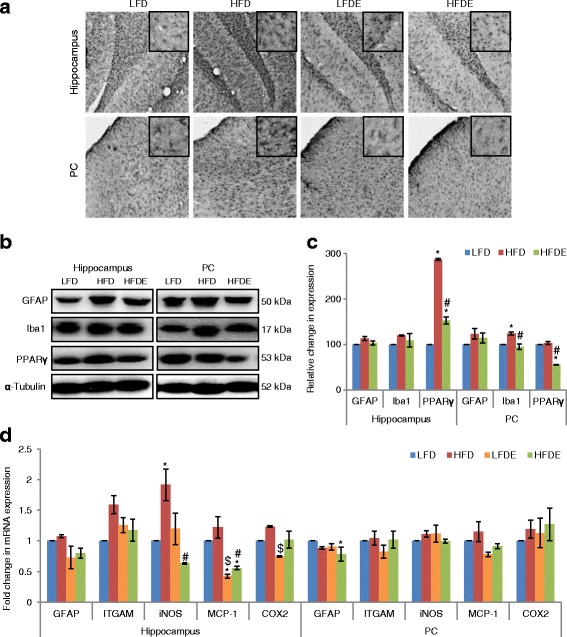
ASH suppressed inflammation at both transcriptional and translational levels. **a** Immunostaining of GFAP in the hippocampus and PC regions of LFD, HFD, LFDE, and HFDE rats (*n* = 3 each group). Insets show high magnification images of GFAP staining. Upregulation of GFAP in HFD rats was seen, which was ameliorated in HFDE rats. **b**, **c** Representative Western blot analysis for GFAP, Iba1, and PPARγ in the hippocampus and PC regions of LFD, HFD, and HFDE animals. Histograms represent percent change in intensity taking value in LFD rats as 100%. **d** Histograms representing fold change in mRNA expression of GFAP, ITGAM, iNOS, MCP-1, and COX2 in the hippocampus and PC regions of rat brain among the four groups of animals (*n* = 3–4 each group). Values are expressed as mean ± SEM. * *p* ≤ 0.05 LFD versus HFD, LFDE, and HFDE rats, ^#^ *p* ≤ 0.05 HFD versus HFDE rats, ^$^ *p* ≤ 0.05 HFD versus LFDE rats, Holm–Sidak method after one-way ANOVA

Glial cells play an indispensable role during generation of immune response under various stressed conditions. High fat diet feeding led to upregulation in the expression of GFAP, an astroglial marker, in both hippocampus and PC regions of the brain (Fig. 2a). Western blotting and real-time PCR data also supported the immunostaining data of upregulation of GFAP in hippocampus and PC regions of HFD animals (Fig. 2b–d). The supplementation of high fat diet with ASH suppressed the change in GFAP expression in HFDE animals as observed by immunostaining, Western blotting, and real-time PCR study (Fig. 2). The expression of GFAP in the LFDE group was not different from the LFD group. These results are suggestive of reactive gliosis in HFD animals, which was effectively suppressed in ASH-fed HFDE animals. Reactive gliosis may be one of the initial events leading to chronic immune activation and inflammatory response. So, we further studied the expression of some inflammatory markers by both Western blotting and real-time PCR.

The expression of Iba1, a microglial cell-specific marker, was upregulated by 19.9% in the hippocampus region (*p* ≤ 0.05) as compared to LFD animals (Fig. 2b, c). The treatment with ASH suppressed the expression of Iba1 in HFDE group. PPARγ, which is a nuclear receptor controlling the transcription of genes responsible for growth and differentiation of adipocytes, showed significant upregulation (*p* ≤ 0.05) in the hippocampus region, whereas its expression was alleviated in the HFDE group (Fig. 2b, c).

Further, the mRNA levels of various inflammatory markers were studied by quantitative real-time PCR (Fig. 2d). ITGAM, which is a gene coding for Cd11b on macrophages/microglia, showed 1.6-fold upregulation in the hippocampus region of HFD animals, which was reduced to 1.2-fold in the HFDE group as compared to LFD animals. The mRNA levels of other inflammatory markers, such as iNOS (inducible nitric oxide synthase), MCP-1 (monocyte chemoattractant protein-1), and COX2 (Cyclooxygenase-2) also showed upregulation in the hippocampus region of HFD animals, which was alleviated in the HFDE group. Overall, the mRNA levels of different markers in the LFDE group were similar to the LFD group.

Further, the expression of Iba1 showed 23.5% increase in the PC region of HFD animals (*p* ≤ 0.05), which was suppressed with ASH supplementation in HFDE animals (Fig. 2b, c). The expression of PPARγ was not different in the PC region between the LFD and HFD group animals; however, it was reduced significantly (*p* ≤ 0.05) in HFDE animals (Fig. 2b, c). However, no difference in the mRNA levels of ITGAM, iNOS, MCP-1, and COX2 was observed in the PC region among the different groups of animals (Fig. 2d).

### ASH modulated the expression of pro-inflammatory cytokines

**Fig. 3:**
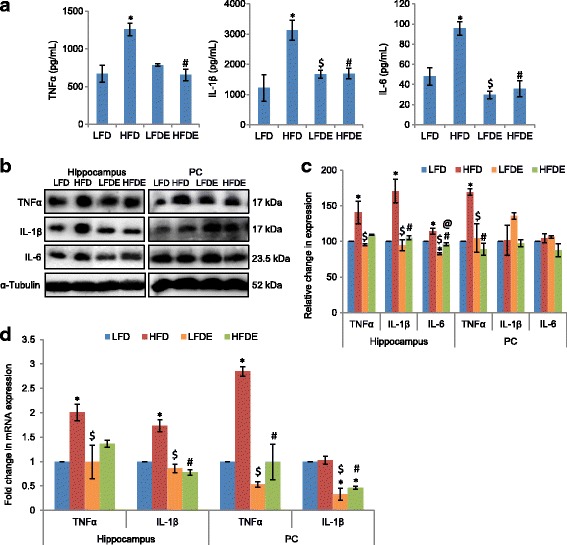
ASH suppressed the synthesis and secretion of pro-inflammatory cytokines. **a** Histograms represent the serum levels of TNFα, IL-1β, and IL-6 among LFD, HFD, LFDE, and HFDE rats (*n* = 8 each group). **b**, **c** Representative Western blot analysis for TNFα, IL-1β, and IL-6 in the hippocampus and PC regions of rat brain among the four groups of animals. Histograms represent percent change in intensity taking value in LFD rats as 100%. **d** Histograms representing fold change in mRNA expression of TNFα and IL-1β in the hippocampus and PC regions of rat brain among the four groups of animals (*n* = 3–4 each group). Values are expressed as mean ± SEM. * *p* ≤ 0.05 LFD versus HFD, LFDE, and HFDE rats, ^#^ *p* ≤ 0.05 HFD versus HFDE rats, ^$^ *p* ≤ 0.05 HFD versus LFDE rats, ^@^ *p* ≤ 0.05 LFDE versus HFDE rats, Holm–Sidak method after one-way ANOVA

Since the preliminary data showed induction of inflammation in HFD animals, we further extended the work to study the expression of pro-inflammatory cytokines TNFα, IL-1β, and IL-6 by ELISA, Western blotting, and quantitative real-time PCR (Fig. 3). In ELISA-based assays, the circulating levels of TNFα, IL-1β, and IL-6 showed significant increase (*p* ≤ 0.001) in the HFD group animals as compared to the LFD group, which were alleviated in the HFDE animals (Fig. 3a). Further, the expression of TNFα, IL-1β, and IL-6 also showed significant increase (*p* ≤ 0.05) in the hippocampus region of the HFD group as compared to the LFD group at both translational (Western blotting Fig. 3b, c) and transcriptional levels (quantitative real-time PCR data Fig. 3d). However, the supplementation of ASH suppressed the HFD-induced increase of pro-inflammatory cytokines in the HFDE group (Fig. 3b–d). The expression of TNFα also showed upregulation in HFD animals as compared to LFD animals in the PC region (*p* ≤ 0.05), but it was reduced to near-control (LFD) level in both LFDE and HFDE animals as is evident from Western blotting (Fig. 3b, c) and real-time PCR results (Fig. 3d). No change was seen in the mRNA expression of IL-1β between the LFD and HFD groups, but it showed significant recovery in the LFDE and HFDE groups in the PC region at transcriptional level (Fig. 3d).

### ASH ameliorated hyperleptinemia and hyperinsulinemia caused by HFD

**Fig. 4:**
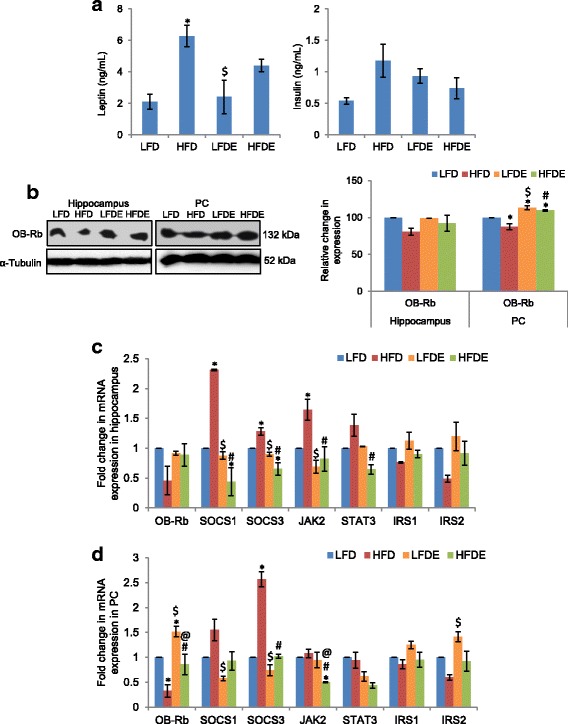
ASH prevented hyperleptinemia and hyperinsulinemia. **a** Histograms represent the serum levels of leptin and insulin among LFD, HFD, LFDE, and HFDE rats (*n* = 8 each group). **b** Representative Western blot analysis for OB-Rb in the hippocampus and PC regions of rat brain. Histograms represent percent change in intensity taking value in LFD rats as 100%. **c**, **d** Histograms representing fold change in mRNA expression of OB-Rb, SOCS1, SOCS3, JAK2, STAT3, IRS1, and IRS2 in the hippocampus (**c**) and PC (**d**) regions of rat brain among the four groups of animals (*n* = 3–4 each group). Values are expressed as mean ± SEM. * *p* ≤ 0.05 LFD versus HFD, LFDE, and HFDE rats, ^#^ *p* ≤ 0.05 HFD versus HFDE rats, ^$^ *p* ≤ 0.05 HFD versus LFDE rats, ^@^ *p* ≤ 0.05 LFDE versus HFDE rats, Holm–Sidak method after one-way ANOVA

Further, we estimated the circulating levels of leptin and insulin in these groups using ELISA-based assays. The level of leptin significantly increased in the HFD group (*p* ≤ 0.01) as compared to LFD animals and was partially recovered in the HFDE group (Fig. 4a, left panel). The level of leptin in the LFDE group was not different from the LFD group. Along with the increase in circulating level of leptin, the expression of leptin receptor OB-Rb showed decrease in HFD animals in both the hippocampus and PC regions of the brain as shown by both Western blot (Fig. 4b) and real-time PCR analysis (Fig. 4c, d), whereas, the expression of OB-Rb in HFDE and LFDE animals was similar to the LFD group. The circulating level of insulin also showed increase in the HFD animals (*p* = 0.06), which was recovered to near-control (LFD) level in HFDE animals (Fig. 4a, right panel).

Since SOCS1 and SOCS3 have been implicated in the development of leptin and insulin resistance, we further evaluated the mRNA expression level of these genes. Real-time PCR analysis showed significant upregulation (*p* ≤ 0.05) of both SOCS1 and SOCS3 in the hippocampus and PC regions of the brain, which was alleviated in HFDE animals (Fig. 4c, d). Leptin is known to activate JAK2-STAT3 signaling pathway, which regulates the expression of SOCS3. So, we further elucidated the differences in mRNA expression of JAK2 and STAT3. The expression of both JAK2 and STAT3 showed significant upregulation (*p* ≤ 0.05) in the hippocampus region of HFD animals, which was ameliorated by ASH in the HFDE group (Fig. 4c). However, the PC region did not show any change in the expression of JAK2 and STAT3 (Fig. 4d). We further studied the mRNA expression level of insulin receptor substrates IRS1 and IRS2. Consistent with the high level of circulating insulin in the serum, the mRNA levels of IRS1 and IRS2 showed downregulation in HFD animals in both the hippocampus and PC regions, which was restored to the normal level with ASH supplementation in HFDE animals (Fig. 4c, d).

### ASH modulated NF-κB pathway and prevented apoptosis

**Fig. 5:**
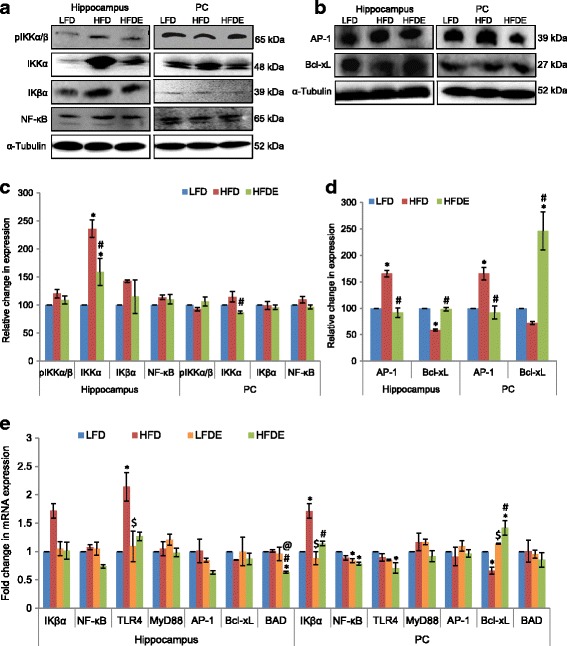
ASH modulated NF-κB pathway and prevented apoptosis. **a**, **c** Representative Western blot analysis for markers of NF-κB pathway in the hippocampus and PC regions of brain among the LFD, HFD, and HFDE animals. Histograms represent percent change in intensity taking value in LFD rats as 100%. **b**, **d** Representative Western blot analysis for apoptotic markers AP-1 and Bcl-xL in the hippocampus and PC regions of brain among the LFD, HFD, and HFDE rats. Histograms represent percent change in intensity taking value in LFD rats as 100%. **e** Histograms representing fold change in mRNA expression of markers of NF-κB pathway and apoptotic markers in the hippocampus and PC regions of rat brain among the four groups of animals (*n* = 3–4 each group). Values are expressed as mean ± SEM. * *p* ≤ 0.05 LFD versus HFD, LFDE, and HFDE rats, ^#^ *p* ≤ 0.05 HFD versus HFDE rats, ^$^ *p* ≤ 0.05 HFD versus LFDE rats, ^@^ *p* ≤ 0.05 LFDE versus HFDE rats, Holm–Sidak method after one-way ANOVA

We further extended the study to elucidate the effect of ASH on NF-κB pathway by both Western blot and quantitative real-time PCR (Fig. 5). The phosphorylated form of IKKα/β showed slight upregulation in the hippocampus region of the HFD group as compared to the LFD group, which was partially reduced with ASH supplementation in the HFDE group (Fig. 5a, c). Further, IKKα, which is known to be activated during CNS insult, showed significant (*p* ≤ 0.05) increase in the hippocampus region of the HFD group animals which was ameliorated in ASH-fed HFDE animals (Fig. 5a, c). IKβα, which is responsible for transcriptional activation of NF-κB, showed upregulation in the hippocampus region of HFD animals in both protein (Fig. 5a, c) and mRNA expression (Fig. 5e), which was suppressed in ASH-fed HFDE animals. However, the PC region of HFD animals showed upregulation only in mRNA expression (Fig. 5e), which was ameliorated in HFDE animals. NF-κB did not show any significant change in hippocampus region among the four groups (Fig. 5a, c, and e). However, its mRNA expression was significantly (*p* ≤ 0.05) downregulated in the PC region of the LFDE and HFDE groups (Fig. 5e). TLR4, which activates NF-κB pathway via MyD88 protein, showed significant (*p* ≤ 0.05) upregulation in the hippocampus region of HFD animals (Fig. 5e), but its expression was alleviated in HFDE animals. TLR4 also showed significant (*p* ≤ 0.05) downregulation in the PC region of HFDE animals (Fig. 5e). MyD88 showed slight upregulation in the HFD animals in the PC region only (Fig. 5e). LFDE animals also showed slight upregulation in the expression of MyD88 in both the hippocampus and PC regions (Fig. 5e) of the brain, but the change was not statistically significant.

Since NF-κB is also known to be instrumental in mediating cell survival pathway, we further studied the expression of apoptotic marker proteins. AP-1 showed significant upregulation in HFD animals as compared to the LFD group in both the brain regions at translational level (Fig. 5b, d), but its expression showed near-control level in ASH-supplemented HFDE animals. However, no change in mRNA expression of AP-1 was observed in HFD animals in either of the brain regions at transcriptional level (Fig. 5e), but it was reduced to 0.6-fold in the hippocampus region in the HFDE group. The PC region did not show any change in mRNA expression of AP-1 (Fig. 5e). Further, Bcl-xL, which is an anti-apoptotic protein, showed downregulation in the HFD animals as compared to LFD animals in both Western blot (Fig. 5b, d) and real-time PCR analysis (Fig. 5e), but the expression of Bcl-xL increased significantly in both the brain regions in LFDE and HFDE animals after supplementation with ASH (Fig. 5b, d, and e). BAD, the pro-apoptotic marker, did not show any change in either of the brain regions (Fig. 5e) in the HFD group, but it was reduced to 0.6-fold in the hippocampus region in the HFDE group. The PC region showed slight upregulation in mRNA expression of BAD in the HFDE group, but it was not statistically significant (Fig. 5e).

### ASH suppressed HFD-induced inflammatory response in the hypothalamus region

**Fig. 6:**
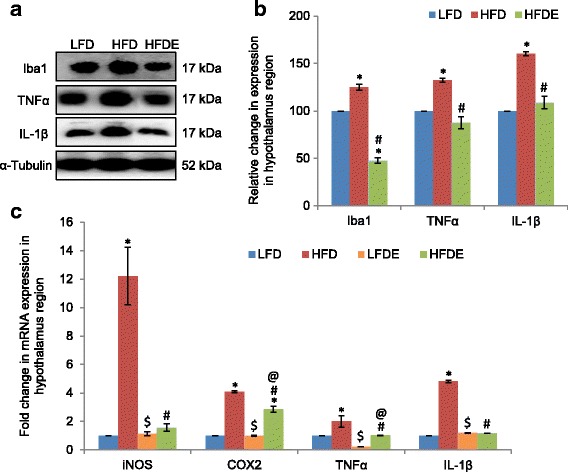
ASH suppressed inflammation caused by DIO in the hypothalamus. **a**, **b** Representative Western blot analysis for Iba1, TNFα, and IL-1β in the hypothalamus region of LFD, HFD, and HFDE animals. Histograms represent percent change in intensity taking value in LFD rats as 100%. **c** Histograms representing fold change in mRNA expression of iNOS, COX2, TNFα, and IL-1β in the hypothalamus region of rat brain among the four groups of animals (*n* = 3–4 each group). Values are expressed as mean ± SEM. * *p* ≤ 0.05 LFD versus HFD, LFDE, and HFDE rats, ^#^ *p* ≤ 0.05 HFD versus HFDE rats, ^$^ *p* ≤ 0.05 HFD versus LFDE rats, ^@^ *p* ≤ 0.05 LFDE versus HFDE rats, Holm–Sidak method after one-way ANOVA

As the hypothalamus is most affected by obesity-associated neuroinflammation, so we also evaluated the expression of some inflammatory markers in this region. The expression of Iba1 showed significant increase by 24.9% in the HFD fed group as compared to the LFD group (Fig. 6a, b), while it was significantly reduced to 47.7% with ASH supplementation in HFDE group animals. The increase in expression of Iba1 in the HFD group was also complemented by significant upregulation in the expression of pro-inflammatory cytokines TNFα and IL-1β (*p* ≤ 0.05) (Fig. 6a, b). However, ASH supplementation effectively suppressed the HFD-induced expression of these cytokines as evident from their near-control expression in the HFDE group.

Further, mRNA expression of iNOS significantly increased by 12.2-fold in the HFD fed group (*p* ≤ 0.05) as compared to LFD fed rats (Fig. 6c), but it was notably reduced to near-control level with ASH supplementation in the HFDE group. The mRNA levels of other inflammatory markers, such as COX2, TNFα, and IL-1β also showed upregulation in the hypothalamus region of HFD fed animals (*p* ≤ 0.05) (Fig. 6c). The transcriptional expression of COX2 was suppressed to some extent only in the HFDE group, but TNFα and IL-1β showed near-control levels with HFD+ASH regimen (Fig. 6c). The mRNA expressions of iNOS, COX2, and IL-1β in the LFDE group were not different from the LFD group, but TNFα showed 78% reduction in the LFDE group animals as compared to the LFD group.

### ASH modulated leptin signaling pathway in hypothalamus

**Fig. 7:**
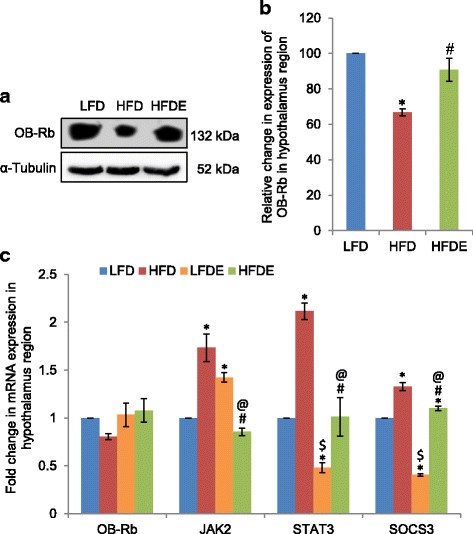
ASH modulated leptin signaling pathway in the hypothalamus. **a**, **b** Representative Western blot analysis for OB-Rb in the hypothalamus region of rat brain. Histogram represents percent change in intensity taking value in LFD rats as 100%. **c** Histograms representing fold change in mRNA expression of OB-Rb, JAK2, STAT3, and SOCS3 in the hypothalamus region of rat brain among the four groups of animals (*n* = 3–4 each group). Values are expressed as mean ± SEM. * *p* ≤ 0.05 LFD versus HFD, LFDE, and HFDE rats, ^#^ *p* ≤ 0.05 HFD versus HFDE rats, ^$^ *p* ≤ 0.05 HFD versus LFDE rats, ^@^ *p* ≤ 0.05 LFDE versus HFDE rats, Holm–Sidak method after one-way ANOVA

Since hypothalamus is the main site for leptin action, we evaluated the effect of HFD on leptin signaling pathway in this region. The expression of OB-Rb, the long form of leptin receptor, showed reduced expression in the HFD fed group as compared to the LFD fed group at both transcriptional (Fig. 7c) and translational (Fig. 7a, b) levels. This also corresponded with the higher circulating level of leptin in this group. However, the expression of OB-Rb was restored to near-control level with ASH supplementation in the HFDE group animals. Since leptin is known to activate JAK2-STAT3-SOCS3 pathway [27], we further elucidated the mRNA expression level of these markers in the hypothalamus region. All the three markers, viz., JAK2, STAT3, and SOCS3, showed significant upregulation in the HFD-fed animals (*p* ≤ 0.05) as compared to the LFD group (Fig. 7c). However, ASH supplementation effectively suppressed the stress-induced expression of these markers. The LFDE group also showed significant reduction in expression of STAT3 and SOCS3 as compared to the LFD group, but expression of JAK2 was enhanced significantly (Fig. 7c).

### ASH modulated NF-κB pathway in hypothalamus

**Fig. 8:**
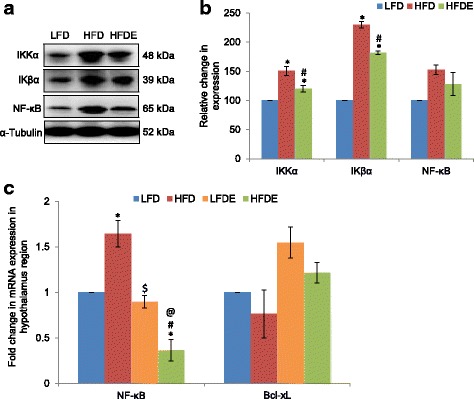
ASH modulated NF-κB signaling pathway in the hypothalamus. **a**, **b** Representative Western blot analysis for markers of NF-κB pathway in the hypothalamus region of the brain among the LFD, HFD, and HFDE animals. Histograms represent percent change in intensity taking value in LFD rats as 100%. **c** Histograms representing fold change in mRNA expression of NF-κB and Bcl-xL in the hypothalamus region of rat brain among the four groups of animals (*n* = 3–4 each group). Values are expressed as mean ± SEM. * *p* ≤ 0.05 LFD versus HFD, LFDE, and HFDE rats, ^#^ *p* ≤ 0.05 HFD versus HFDE rats, ^$^ *p* ≤ 0.05 HFD versus LFDE rats, ^@^ *p* ≤ 0.05 LFDE versus HFDE rats, Holm–Sidak method after one-way ANOVA

Considering the significant inflammatory changes observed in the hypothalamus region of the HFD group animals, we further analyzed the expression level of NF-κB pathway marker proteins. The upstream markers in NF-κB signaling pathway, IKKα and IKβα, showed significant upregulation by 50 and 130%, respectively, in HFD group animals as compared to the LFD group (Fig. 8a, b). The expression of these marker proteins was attenuated to some extent in the HFDE group. NF-κB also showed upregulated expression in the HFD fed group at both transcriptional (Fig. 8c) and translational (Fig. 8a, b) levels. However, it was only reduced to some extent at the translational level (Fig. 8a, b), but mRNA level of NF-κB showed significant reduction in the HFDE group (Fig. 8c). The expression of NF-κB in the LFDE group was similar to the LFD group. Further, the mRNA level of Bcl-xL, the anti-apoptotic marker protein, showed 24% reduction in the HFD-fed group as compared to the LFD group (Fig. 8c), but it was restored to near-control level in the HFDE group. The LFDE group animals also showed higher expression of Bcl-xL as compared to the LFD group animals.

## Discussion

The current study provides the scientific validation for anxiolytic and anti-neuroinflammatory properties of dry leaf powder of *W*. *somnifera*. Chronic consumption of high-fat diet has been closely linked to anxiety and depression-like behavior [11, 28]. In the current study, 12 weeks of HFD regimen led to diet-induced obesity as validated from highest body weight of HFD animals (Fig. 1a) and higher calorie intake as compared to the LFD group animals (Fig. 1b) over the 12-week period. HFD regimen initiated in young adult rats induced anxiety-like behavior as is evident from the EPM data (Fig. 1c–f). However, co-supplementation of ASH along with high fat diet in the HFDE group was seen to be instrumental in amelioration of anxiety-like behavior induced by HFD regimen alone. Maximum time spent by the HFD animals in the closed arm and least number of entries and crossings in the open arm are suggestive of the higher anxiety levels in these animals as compared to the LFD group. Also, the number of head dips, i.e., the frequency with which the animal lowered its head over the sides of open arm or from central open area, was least in the HFD group. All these parameters are suggestive of higher anxiety and less exploratory behavior in HFD animals. On the other hand, the number of head dips and time spent by the HFDE animals in either of the arms was comparable to that of the control (LFD) animals. Further, the higher number of entries and crossings, though in the closed arm, are suggestive of the more exploratory behavior of HFDE animals as compared to the other groups, which is indicative of the anxiolytic potential of ASH. A recent study with HFD regimen for 16 weeks has also shown induction of anxiety and anhedonia behavior in rats [29]. Anxiety disorders have also been shown to be prevalent in obese human subjects [30, 31]. In a recent study from our lab, ASH supplementation along with high fat diet was shown to improve locomotor coordination and prevention of memory-related cognitive impairments as analyzed by rotarod performance test, narrow-beam walk test, and novel object recognition test [32].

HFD-induced obesity has been reported to cause neuroinflammation in the PC [33], hippocampus [34], and hypothalamus [35, 36] regions of the brain. So, we have selected these regions to study the effect of ASH supplementation. The current data suggests that HFD regimen induced reactive gliosis as is evident from marked increase in the expression of GFAP (an astrocytic marker) in both the hippocampus and PC regions of the brain (Fig. 2). Reactive gliosis was also accompanied by microgliosis as is evident from significant increase in the expression of Iba1 (a microglial marker) in the hippocampus, PC (Fig. 2b–d), and hypothalamus (Fig. 6a, b) regions of the brain. Reactive gliosis is a CNS-specific process of recruitment, proliferation, and morphological transformation of astrocytes and microglia in response to brain injury [37]. Inflammation resulting from dietary insults has often been attributed to the activation of microglial cells which increase the synthesis and release of an array of pro-inflammatory cytokines such as IL-16 and IL-22 [4, 38]. Moreover, cytokines have been regarded as the effectors of gliosis, which are known to activate astrocytic cells and cause reactive gliosis by their hyperactivity. In the current study also, microgliosis was complemented with upregulated expression of pro-inflammatory cytokines TNFα, IL-1β, and IL-6 at both transcriptional and translational levels (Figs. 3b–d and 6a–c). The more pronounced changes were seen in TNFα and IL-1β expression in the three brain regions. In circulation also, TNFα, IL-1β, and IL-6 had shown marked increase in the serum samples from the HFD group (Fig. 3a). The reduction in the serum level, protein, and mRNA expression of TNFα, IL-1β, and IL-6 was observed with ASH supplementation in the HFDE group. The inflammation caused by HFD was also evident from the upregulated mRNA expression of iNOS, MCP-1, and COX2 (Figs. 2d and 6c) in the three brain regions but with more pronounced changes in the hypothalamus region (Fig. 6c). The hepatic expression of iNOS has been shown to increase in the rats fed with high-fat diet [39, 40] and has been attributed to the occurrence of apoptosis [41]. Further, it has also been demonstrated that pro-inflammatory cytokines are directly involved in the upregulation of iNOS [40]. Based on the current results, it may be suggested that the upregulation of TNFα and IL-1β may be responsible for the higher mRNA levels of iNOS in HFD animals.

Further, MCP-1 is known to recruit monocytes into the adipose tissue in obese subjects and enhance obesity-associated chronic inflammation and insulin resistance [42]. Thus, the upregulation of MCP-1 in the hippocampus and PC regions of the HFD group (Fig. 2d) may have contributed to the inflammatory phenotype in these discrete brain regions, which may also be the factor for upregulation of pro-inflammatory cytokines. The supplementation of bamboo extract has been shown to lower the circulating levels of MCP-1, when supplemented in high fat diet for 6 months [43]. The upregulation of MCP-1 in our study also correlated with the upregulation of PPARγ in the hippocampus region of HFD rats (Fig. 2b, c). PPARγ is a protein which is known to regulate the differentiation of adipose tissue [44]. Further, COX2, another important indicator of inflammation, was also upregulated in the three brain regions of HFD rats (Figs. 2d and 6c). However, high fat diet supplemented with ASH seemed to suppress the HFD-mediated inflammation as observed by significant downregulation of PPARγ in HFDE rats and near-control mRNA expression of iNOS, MCP-1, and COX2 in these rats.

The inflammatory response from the gut is traversed to the brain via the parasympathetic nervous system in a vagus-specific mechanism (reviewed by Das, [45]). Upon ingestion of energy-dense food, the gut initiates a series of homeostatic mechanisms to regulate plasma glucose levels, thereby enhancing the insulin secretion. Adipose tissue infiltrates macrophages and lymphocytes which secrete increased amounts of TNFα and IL-6, leading to systemic inflammation. Leptin secretion is also enhanced in the adipose tissue and stomach. Further, *Firmicutes*, the predominant bacteria in the gut of obese animals [45], break down the polysaccharides leading to their digestion and absorption into the adipose tissue, which causes obesity. The long chain fatty acid-Coenzyme A (LCFA-CoA), resulting from breakdown of saturated fatty acids, is sensed by the gut, which signals the brain through the vagus nerve via the hindbrain. LCFA-CoA triggers counter-regulatory responses in the hypothalamus and gut to regulate food intake and normalize plasma glucose concentrations. However, continuous ingestion of energy-dense foods disrupts this regulatory system leading to obesity by increasing hunger signals. The peripheral inflammatory signals reach the brain through leaky blood–brain barrier and cause inflammation in the CNS.

The inflammatory milieu and cytokine dysregulation have been reported to lead to depression, aberrant behavior, and cognitive decline accompanied by anxiety [46]. The expression of various cytokines and costimulatory/adhesion molecules is known to promote the generation of reactive oxygen and nitrogen species, thus leading to oxidative stress [47, 48] and aberrant behavior in animals [49]. Thus, higher rate of inflammation and upregulated synthesis and release of pro-inflammatory cytokines may be responsible for the anxiety-like behavior observed in the HFD animals in the EPM test (Fig. 1b–e). ASH supplementation ameliorated the inflammation and anxiety in HFDE animals. The root extract of *W*. *somnifera* has been shown to ameliorate anxiety-like behavior induced by hypobaric hypoxia in rats [50]. Also, the anti-inflammatory activity of methanol:water (3:7) extract from the leaves of *W*. *somnifera* has been reported in stainless steel implant-induced inflammation in zebrafish [51]. The anti-inflammatory activity of the plant has been attributed to the rich concentration of flavonoids and phenolic acids such as rutin, gallic acid, quercetin, vanillic acid, and kaempferol in the TLC-separated portion of the supernatant of methanolic extract from the leaves of the plant [51].

Our lab has recently reported the anti-neuroinflammatory potential of water extract from leaves of *W*. *somnifera* (ASH-WEX) in cell culture-based model system using LPS (lipopolysaccharide)-induced primary microglial cells and BV-2 murine microglial cell line [21]. ASH-WEX was found to inhibit the production of reactive oxygen and nitrogen species, thereby preventing the oxidative damage occurring due to LPS-induced neuroinflammation. ASH-WEX was also found to be instrumental in suppressing the synthesis and release of pro-inflammatory cytokines TNFα, IL-1β, and IL-6 in both the model systems. The translocation of NF-κB from the cytoplasm into the nuclear compartment was inhibited, which is a crucial step for the expression of various cytokines, chemokines, and MHCs in the microglia in response to a CNS insult. ASH-WEX was found to pose its anti-inflammatory effects by the induction of apoptosis in inflamed microglia which is one of the proposed mechanisms for the anti-inflammatory activity of compounds to inhibit neuroinflammation [52]. The anti-neuroinflammatory activity of ASH-WEX in this study has been attributed to the active components, withaferin A and withanone.

Further, the HFD animals in our study also showed high circulating levels of leptin, which was partially suppressed in the ASH fed animals (Fig. 4a, left panel). Hyperleptinemia has been associated with diet-induced obesity, and the development of leptin resistance in obesity has been attributed to the downregulation of cellular responses to leptin [53]. High level of fat mass may be responsible for the higher leptin levels observed in HFD and HFDE animals as compared to the LFD group. Corresponding to the circulating level of leptin, HFD animals weighed more than the HFDE animals over the period of 12 weeks (Fig. 1a). HFD regimen also caused significant increase in the levels of cholesterol and triglycerides as compared to the LFD group, and supplementation of diet with *W*. *somnifera* suppressed HFD-induced hypercholesterolemia and hypertriglyceridemia (data not shown). In another study, the ineffectiveness of *Orthosiphon stamineus* plant for reducing weight in diet-induced obesity has been linked to the development of leptin resistance in mice [54]. Further, consistent with the high circulating level of leptin in HFD rats, corresponding decrease in the expression of leptin receptor OB-Rb was also observed in these animals (Figs. 4b–d and 7a–c). Furthermore, SOCS3 showed significant upregulation in the three brain regions in HFD rats of our study along with upregulation of JAK2 and STAT3 in the hippocampus and hypothalamus regions of HFD rats, but supplementation with ASH was found to suppress the HFD-induced upregulation of JAK2, STAT3, and SOCS3 genes (Figs. 4c, d and 7c). SOCS3 has also been linked to the development of leptin resistance during obesity [55]. Bjorbaek et al. [56] showed the induction of SOCS3 expression mediated by leptin. The high circulating levels of leptin during obesity lead to the chronic activation of intracellular JAK-STAT3 signaling, which further induces the expression of SOCS3 [27]. SOCS3 acts as an inhibitor of leptin signal transduction pathway by preventing the phosphorylation of JAK2 and STAT3, which is an important step for signal transmission on leptin receptors [57]. The higher mRNA expression of SOCS3 in our study also supports the development of leptin resistance, which may be responsible for high level of circulating leptin and reduction in the expression of OB-Rb in HFD rats. ASH supplementation, however, was observed to normalize the level of JAK2, STAT3, SOCS3, and OB-Rb expression in HFDE rats.

Further, the HFD animals also developed insulin resistance as indicated by the higher circulating levels of insulin in this group (Fig. 4a, right panel). Consumption of fat-rich diet for 16 weeks has been shown to induce insulin resistance in the hypothalami of Wistar rats [58]. Both SOCS1 and SOCS3 have been attributed to the development of insulin resistance [59] by inhibiting the phosphorylation of IRS1 and IRS2 proteins. Inhibition of SOCS1 in diabetic and obese mice has been shown to improve insulin sensitivity [60]. Corresponding with the high circulating level of insulin in the HFD group of our study, the mRNA level of SOCS1 was also higher in the hippocampus and PC regions of the brain (Fig. 4c). Further, the mRNA level of insulin receptor substrates IRS1 and IRS2 was downregulated in HFD animals (Fig. 4c), which corresponded with the high circulating level of insulin in these animals. Additionally, the insulin resistance observed in HFD rats also corresponded with the upregulated mRNA expression of MCP-1 in both the brain regions (Fig. 2d), which is also responsible for the development of insulin resistance. The upregulation of SOCS1 and SOCS3 has been projected as a mechanism for imparting insulin resistance by the downregulation of IRS1 [61]. On the other hand, ASH supplementation in high fat diet was observed to normalize the insulin levels by restoring the mRNA levels of SOCS1 (Fig. 4c). Recently, the withanolides from *W*. *somnifera* have been shown to impart hypoglycemic activity in in vitro model system [62].

As NF-κB pathway is considered as one of the major regulators of inflammation, so we further extended our study to elucidate the NF-κB pathway (Figs. 5 and 8). Saturated fatty acids are known to produce an inflammatory response through the activation of TLR4 signaling in the hypothalamus region [63]. TLR4 is known to activate NF-κB pathway via MyD88 protein. Further, IKβα is an inhibitory regulator of NF-κB transcriptional activation. Upon stimulus with cytokines or endotoxins, the upstream kinases IKKα and IKKβ get activated and phosphorylate IKβα, leading to its proteasomal degradation and transcriptional activation of NF-κB. In our study, TLR4, IKKα, and IKβα showed significant upregulation in HFD animals (Fig. 5a, c, and e). The inflammatory milieu in HFD animals may cause the activation of TLR4 and IKKα, which showed significant upregulation in the hippocampus region. Significant upregulation in expression of IKKα was observed in the hypothalamus region as well (Fig. 8a, b). Overnutrition has been reported to activate hypothalamic IKKβ/NF-κB through endoplasmic reticulum stress in the hypothalamus region, which leads to the interrupted insulin and leptin signaling [64]. In our study, the expression of total IKβα also showed upregulation at both transcriptional and translational levels (Figs. 5a, c, and e and 8a, b). It may be suggested that IKKα acted in an NF-κB independent mechanism. IKKα has been implicated in the ROS-mediated apoptosis by modulating the transcriptional activity of p53 [65]. In our study, HFD regimen also led to the induction of apoptosis as is evident from the upregulated translational expression of AP-1 and reduced expression of Bcl-xL (anti-apoptotic protein) at both transcriptional and translational level in the hippocampus and PC regions (Fig. 5b, d and e). Downregulation of Bcl-xL was also evident in the hypothalamus region (Fig. 8c). It may be suggested that HFD-induced apoptosis may be mediated by IKKα in an NF-κB independent mechanism. However, the consumption of ASH seems to reverse the apoptosis as is evident from increase in expression of Bcl-xL and normalization of AP-1 expression along with amelioration of IKKα. The anti-apoptotic activity of *W*. *somnifera* has also been reported by our lab [22] as well as others [66].

Our lab studies are focused on scientific validation of the traditional use of leaf of *W*. *somnifera* using different cell-based and animal model systems. The basic principle of traditional medicine system relies on the wholistic approach towards overall well-being. We have done extensive fractionation studies on *W*. *somnifera* and tested the individual fractions and combinations in cell-based culture model system (unpublished work). The activity of crude extracts was found to be the best, so we have used the crude dry leaf powder of the plant in the current study.

## Conclusions

Owing to its rejuvenating properties, *W*. *somnifera* has been used since time immemorial in the traditional medicine system. The present data suggests that HFD regimen led to anxiety-like behavior and dysregulation of inflammatory molecules. However, ASH supplementation was instrumental in reducing obesity-associated anxiety by modulating the key inflammatory molecules and suppressing apoptosis. Reduction and normalization of GFAP, Iba1, PPARγ, and inflammatory cytokines by ASH further supports its anti-inflammatory potential. ASH also ameliorated the levels of circulating leptin and insulin. The present day lifestyle habits and work schedules have been a common causal factor for high prevalence of obesity in developing and developed nations. Natural therapies to prevent obesity-associated brain dysfunction are limited. ASH offers a novel therapeutic approach to attenuate obesity-associated neuroinflammation. The current study provides the scientific validation to the anxiolytic, anti-inflammatory, and anti-apoptotic properties of leaf powder of the important medicinal plant, *W*. *somnifera*, which may be recommended as a suitable intervention to prevent/slow down the adverse effects of obesity and its associated co-morbid conditions.

## Abbreviations

AP-1: Activator Protein-1
ASH: Dry leaf powder of *Withania somnifera* (Ashwagandha)
Bcl-xL: B-cell lymphoma—extra large
CNS: Central nervous system
COX2: Cyclooxygenase-2
DIO: Diet-induced obesity
EPM: Elevated plus maze
GFAP: Glial fibrillary acidic protein
HFD: High fat diet
HFDE: High fat diet plus ASH extract
Iba1: Ionized calcium-binding adaptor molecule 1
IL-1β: Interleukin-1β
IL-6: Interleukin-6
iNOS: Inducible nitric oxide synthase
IRS: Insulin receptor substrate
JAK2: Janus kinase 2
LFD: Low fat diet
LFDE: Low fat diet plus ASH extract
MCP-1: Monocyte chemoattractant protein-1
MyD88: Myeloid differentiation primary response gene 88
NF-κB: Nuclear factor-kappa B
PC: Piriform cortex
PPARγ: Peroxisome proliferator-activated receptor gamma
SEM: Standard error mean
SOCS: Suppressor of cytokine signaling
STAT3: Signal transducer and activator of transcription 3
TLR4: Toll-like receptor 4
TNFα: Tumor necrosis factor α
